# X-ray fluorescence holography under high-pressure conditions

**DOI:** 10.1107/S1600577525005284

**Published:** 2025-07-18

**Authors:** Xinhui Zhan, Naoki Ishimatsu, Koji Kimura, Naohisa Happo, Halubai Sekhar, Tomoko Sato, Nobuo Nakajima, Naomi Kawamura, Kotaro Higashi, Oki Sekizawa, Hirokazu Kadobayashi, Ritsuko Eguchi, Yoshihiro Kubozono, Hiroo Tajiri, Shinya Hosokawa, Tomohiro Matsushita, Toru Shinmei, Tetsuo Irifune, Koichi Hayashi

**Affiliations:** a Graduate School of Advanced Science and Engineering Hiroshima University Higashi-Hiroshima739-8526 Japan; b Geodynamics Research Center, PIAS Ehime University Matsuyama790-8577 Japan; c Department of Physical Science and Engineering Nagoya Institute of Technology Nagoya466-8555 Japan; d Graduate School of Information Sciences Hiroshima City University Hiroshima731-3194 Japan; e Institute of Industrial Nanomaterials Kumamoto University Kumamoto860-8555 Japan; f Institute of Materials Structure Science High Energy Accelerator Research Organization, KEK Tsukuba305-0801 Japan; g Japan Synchrotron Radiation Research Institute, SPring-8 Sayo679-5198 Japan; h Graduate School of Science University of Hyogo Ako678-1297 Japan; i Research Institute for Interdisciplinary Science Okayama University Okayama700-8530 Japan; j Faculty of Materials for Energy Shimane University Matsue690-8504 Japan; k Graduate School of Science and Technology Nara Institute of Science and Technology Ikoma630-0192 Japan; ESRF – The European Synchrotron, France

**Keywords:** X-ray fluorescence holography, high pressure, SrTiO_3_

## Abstract

This study reports the first application of X-ray fluorescence holography (XFH) under high-pressure conditions. We integrated XFH with a diamond anvil cell to investigate the local structure around Sr atoms in single-crystal SrTiO_3_ under high pressure. By utilizing nano-polycrystalline diamond anvils and a yttrium filter, we effectively eliminated significant background noise from both the anvils and the gasket. This optimized experimental configuration enabled the measurement of Sr *K*α holograms of the SrTiO_3_ sample at pressures up to 13.3 GPa. The variation of lattice constants with pressure was calculated by the shifts of Kossel lines, and real-space images of the atomic structures were reconstructed from the Sr *K*α holograms at different pressures. This work successfully demonstrates the feasibility of employing XFH under high-pressure conditions as a novel method for visualizing pressure-induced changes in the three-dimensional local structure around the specified element.

## Abstract 2

This study demonstrates the first application and feasibility of X-ray fluorescence holography under high-pressure conditions.

## 1. Introduction

X-ray fluorescence holography (XFH) provides a 3D view of the atomic arrangement surrounding a specific element that emits fluorescent X-rays. XFH is defined as an interference image resulting from the fluorescent X-ray from the central atom with those scattered by its neighbouring atoms (Hayashi *et al.*, 2012 ▸). The technique was first proposed in 1986 (Szöke, 1986 ▸) and later experimentally demonstrated in 1996 (Tegze & Faigel, 1996 ▸). In the past three decades, the measurement time for XFH has been reduced considerably from a few months (Tegze & Faigel, 1996 ▸; Kawai *et al.*, 1998 ▸) to as short as several hours or less, facilitating its widespread application across various fields of materials science, including superconductors (Kudo *et al.*, 2019 ▸; Happo *et al.*, 2024 ▸), ferroelectrics (Nakashima *et al.*, 2024 ▸) and soft matter (Ang *et al.*, 2020 ▸). Moreover, the temperature range available for experiments has been extended from room temperature down to cryogenic temperatures as low as 4 K (Hayashi *et al.*, 2021 ▸).

Unlike conventional structural measurement techniques such as X-ray and neutron diffraction, XFH uniquely provides detailed 3D insights into the local structure surrounding the X-ray absorbing atom. Although extended X-ray absorption fine structure spectroscopy can reveal the local structure near the absorbing atom, the structural information is inherently confined to a one-dimensional atomic distribution along the radial direction. In contrast, XFH is capable of reconstructing the 3D local structure within the nanometre scale without prior theoretical models, if the hologram is obtained on the entire Ewald sphere. Consequently, XFH is expected to serve as a new probe for visualizing 3D structural changes under extreme conditions, such as high pressure. However, its application under high-pressure conditions has not yet been established.

There are two methods for recording holograms: the inverse mode and the normal mode (Hayashi *et al.*, 2012 ▸). Recent studies have predominantly employed the inverse mode because it can significantly reduce artefacts in the reconstructed images by recording multiple holograms at varying incident X-ray energies (Barton, 1991 ▸; Hayashi *et al.*, 2012 ▸). However, the inverse mode necessitates rotating the sample about its crystal axis during measurement, a process that is impractical when using a diamond anvil cell (DAC) owing to the limited aperture of its optical windows. In contrast, normal-mode XFH can be performed under fixed-sample conditions. Therefore, normal mode is ideally suited for recording holograms under high-pressure conditions if wide-aperture DACs are available.

This work demonstrates the feasibility of XFH under high-pressure conditions as an innovative technique for visualizing pressure-induced changes in the 3D atomic structure. To achieve this, new DACs were designed for high-pressure XFH measurements and SrTiO_3_ was selected as the measurement sample. SrTiO_3_ is a multifunctional oxide material with a perovskite structure. Previous studies have reported that SrTiO_3_ undergoes a phase transition from cubic to tetragonal around 10 GPa at room temperature. Furthermore, a good single-crystalline sample is commercially available. Therefore, SrTiO_3_ is a suitable sample to examine the feasibility of XFH measurements under high-pressure conditions. By using an X-ray microbeam available at SPring-8, we conducted normal-mode XFH measurements on a SrTiO_3_ single-crystal sample, and optimized the experimental methods and data-collection techniques for high-pressure XFH experiments. After data acquisition and background removal, we successfully obtained Sr *K*α hologram patterns of the SrTiO_3_ crystal in the DAC. Real-space images of the atomic structures of the SrO and TiO_2_ planes under varying pressures were also reconstructed.

## 2. Experimental setup

**Figure 1:**
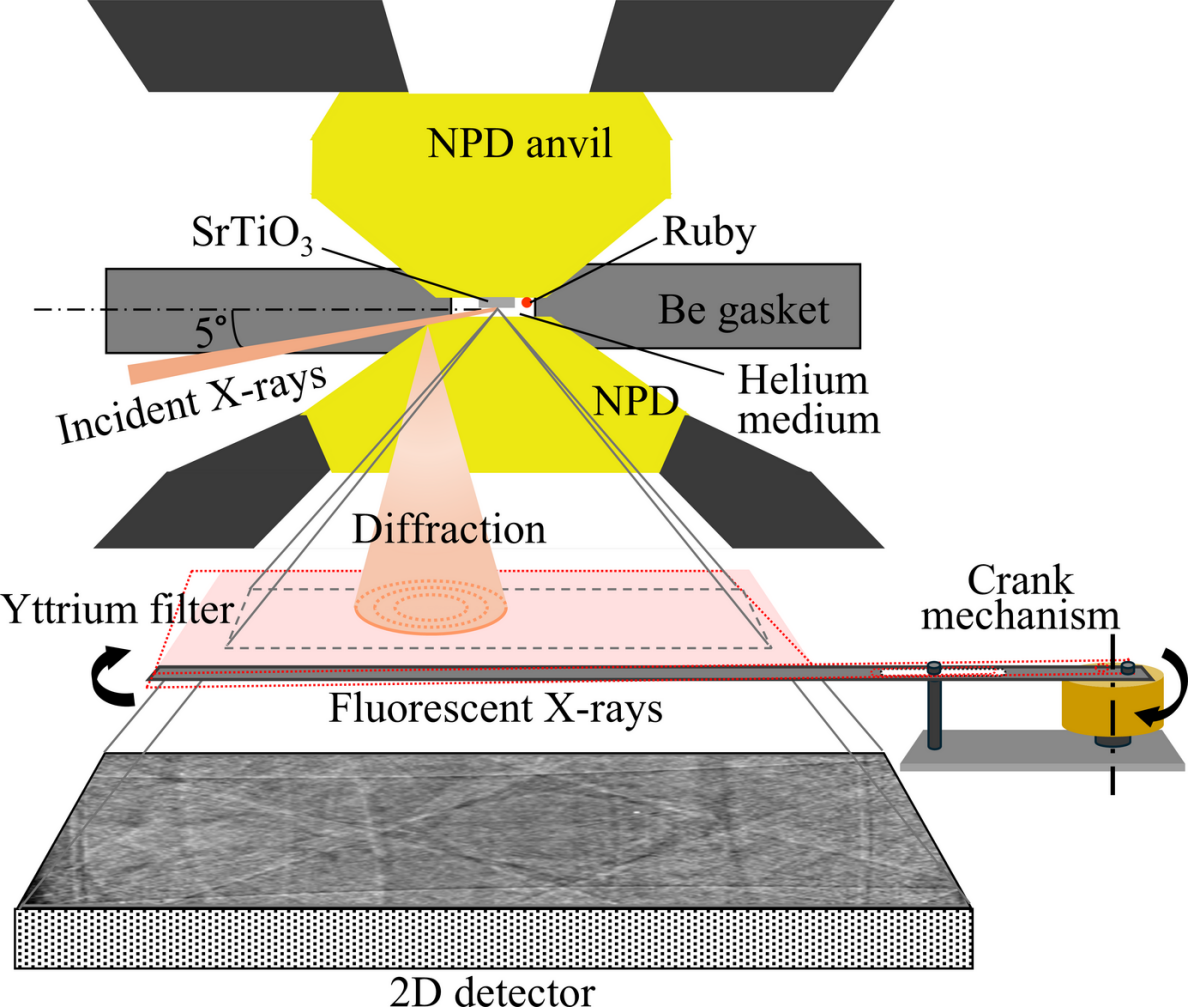
Schematic of the experimental setup for XFH under high-pressure conditions.

**Figure 2:**
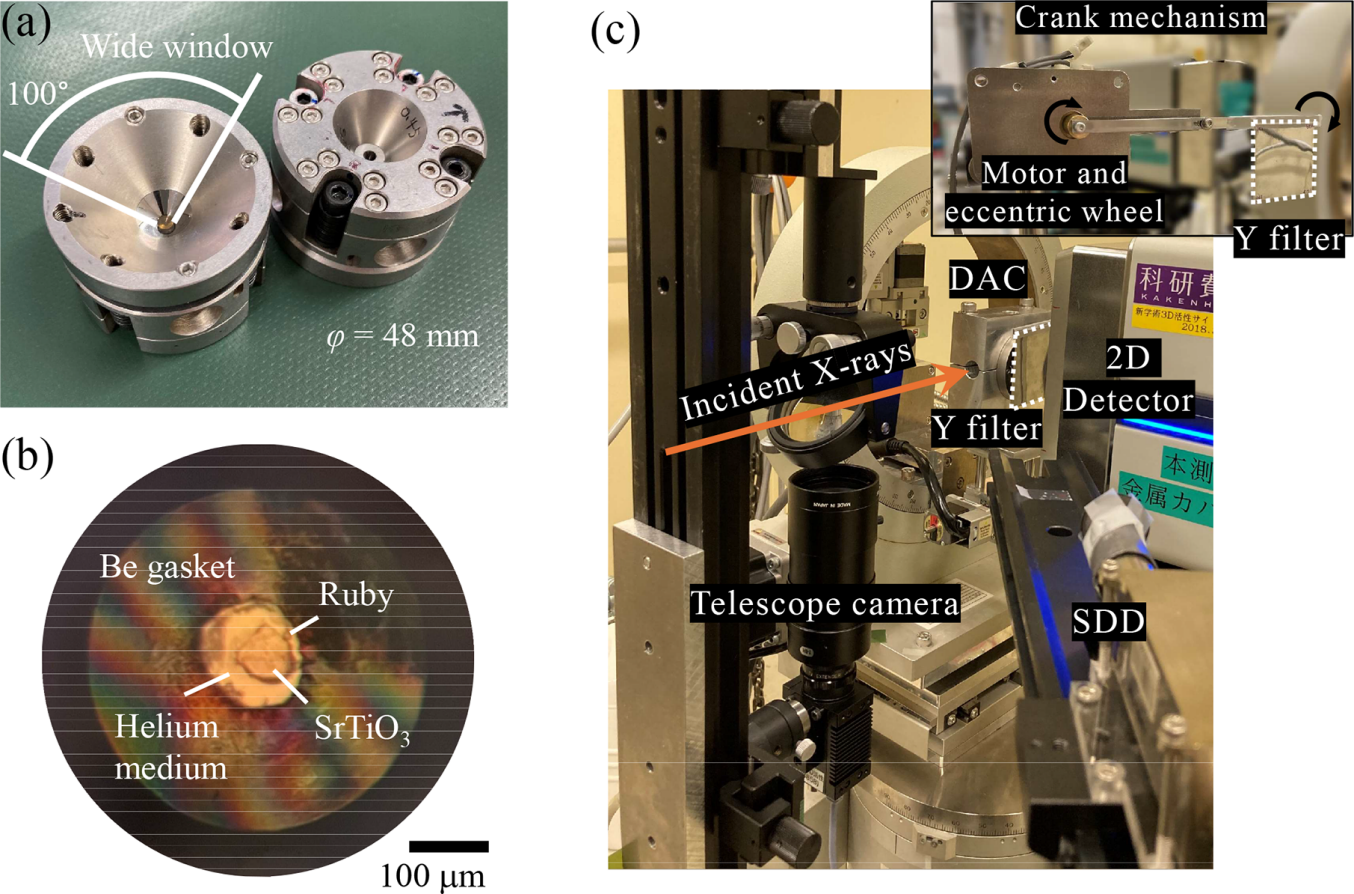
(*a*) Symmetric DAC dedicated to XFH under high pressure. (*b*) Photograph of the sample chamber under 13.3 GPa. (*c*) Photograph of the experimental setup for high-pressure XFH. The inset shows a photograph of the crank mechanism used to reciprocate the Y filter. The filter was connected to an eccentric wheel of the motor by a rod.

Fig. 1 ▸ shows a schematic of our experimental setup for XFH measurements under high pressure. A new symmetric DAC was specifically designed for XFH detection, equipped with a wide window with an aperture angle of 100° on one side [Fig. 2 ▸(*a*)]. This wide window is realized by combining an anvil seat with a Boehler–Almax-type anvil (Boehler & De Hantsetters, 2004 ▸), which maximizes the area over which the XFH image can be collected by the detector. Nano-polycrystalline diamonds (NPDs) (Irifune *et al.*, 2003 ▸) were employed as anvils, with a culet size of 450 µm. The beryllium gasket was pre-compressed to a thickness of 47 µm, and a hole with a diameter of 220 µm was drilled using an electric-discharge machine. Pressurized helium fluid was loaded into the sample chamber and used as the pressure-transmitting medium (Takemura *et al.*, 2001 ▸). Fig. 2 ▸(*b*) shows a photograph of the sample chamber in the DAC at 13.3 GPa. The generated pressure was calibrated using the fluorescence of a ruby ball (Mao *et al.*, 1986 ▸). A commercially available SrTiO_3_ single crystal, with mirror-polished (001) surfaces, was prepared as the sample. To maintain a flat surface for X-ray excitation, only one side of the SrTiO_3_ sample was polished, reducing its thickness to ∼15 µm, before it was cut into smaller plates for loading into the DAC. The final sample plate measured 70 µm × 70 µm, with a thickness of 13 µm. The remaining mirror-polished (001) surface was directed towards the detector and used as the excitation surface.

XFH measurements were conducted in normal mode at the undulator beamlines BL37XU (Nitta *et al.*, 2023 ▸) and BL39XU (Suzuki *et al.*, 2013 ▸) of the SPring-8 synchrotron radiation facility. Fig. 2 ▸(*c*) shows a photograph of the high-pressure XFH setup at BL37XU. The incident X-ray beam was focused to 1.13 µm × 0.68 µm using Kirkpatrick–Baez mirrors, and its energy was tuned to 17.391 keV to excite the Sr *K*α line (14.165 keV). This energy is higher than both the *K*-absorption edge of Sr (16.105 keV) and that of Y (17.038 keV). Incident X-rays penetrated the beryllium gasket and irradiated the (001) surface of SrTiO_3_ in the DAC at a glancing angle of 5°, as shown in Fig. 1 ▸. The DAC was mounted at the centre of a three-circle goniometer stage. A camera and pinhole mirror were placed in the direction of the incident X-rays to roughly align the sample position with the X-rays, while a silicon drift detector (SDD) was used for fine alignment of the sample by monitoring the X-ray fluorescence intensity. A 2D detector (HyPix-9000, Rigaku) acquired the hologram images from the sample. The position of the detector remained fixed relative to the sample throughout the measurement, which is highly effective in collecting fluorescence signals from various solid angles. The detector has an area of 78 mm × 122 mm, with each pixel measuring 100 µm × 100 µm. A polished yttrium (Y) foil with a thickness of 125 µm was inserted between the DAC and the 2D detector to serve as a high-energy cut-off filter. As shown in Fig. 1 ▸, the Y foil was reciprocated by a crank mechanism to maximize function of the high-energy cut-off filter. The filter was connected to an eccentric point of the motor wheel by a rod. When the motor rotates, the wheel drives the connecting rod and filter to swing around the fixed point. All experiments were conducted at room temperature.

## 3. Data collection and processing

**Figure 3:**
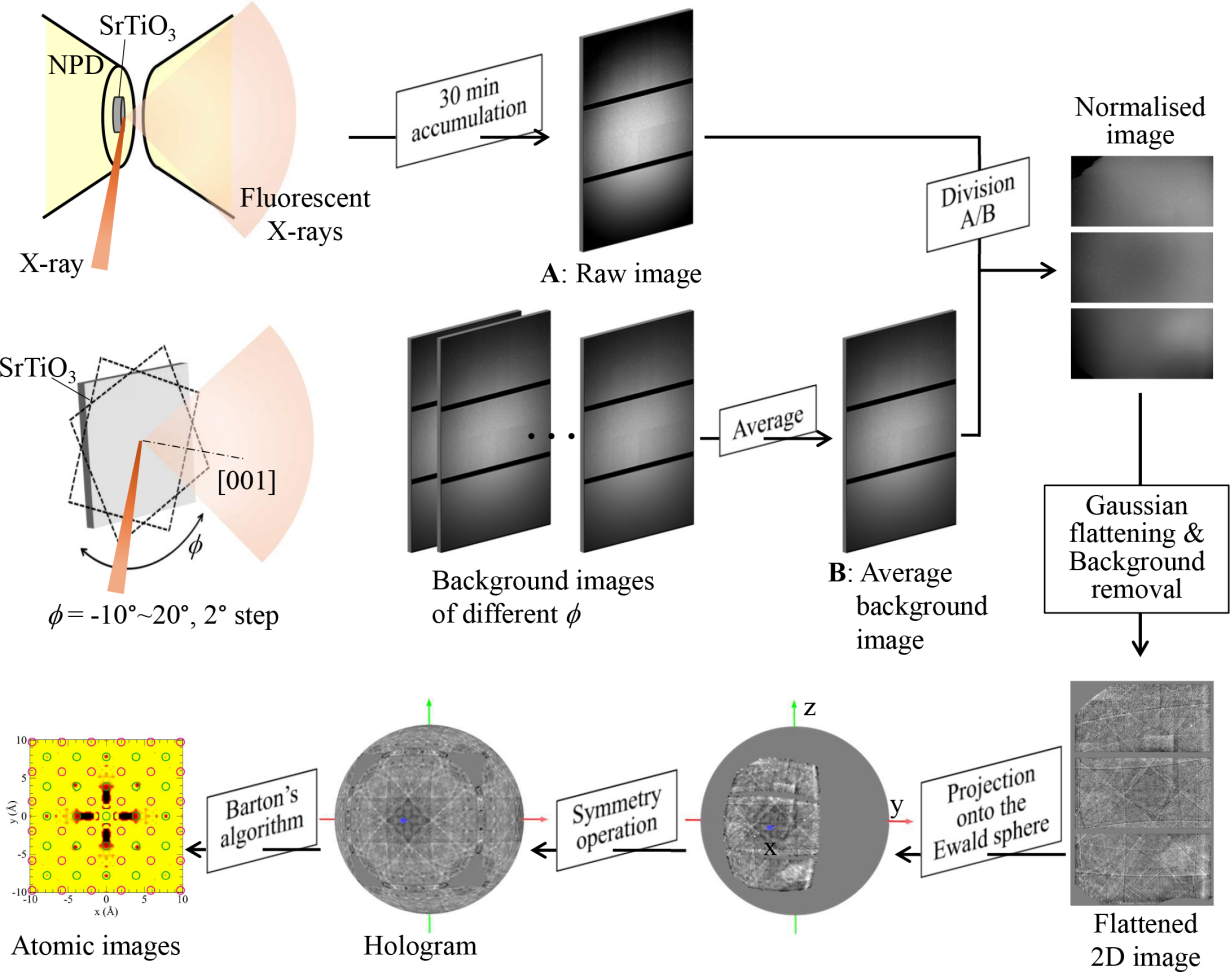
Schematic of data collection and processing for high-pressure XFH experiments.

Fig. 3 ▸ shows the data-processing procedures applied to the sample in the DAC. Measurements were recorded over 30 min at each pressure. Each acquired image was divided by a background image to normalize the different sensitivity of the pixels in the 2D detector. The background image was generated by averaging images of SrTiO_3_ obtained while rotating the sample from ϕ = −10° to 20° in 2° increments about the [001] axis. The background image was collected at ambient pressure without the DAC. After applying Gaussian flattening and removing areas that did not contain hologram patterns, a flattened 2D hologram image was obtained. This image was then projected onto the Ewald sphere surface in *k* space, following procedures described elsewhere (Hayashi, 2023 ▸; Ang *et al.*, 2018 ▸). Analysis of the crystal orientation and the application of appropriate symmetry operations expanded the limited region of the hologram image to cover the entire sphere surface. Finally, the atomic image was reconstructed using Barton’s algorithm (Barton, 1988 ▸). These operations were implemented using the *3D-AIR-IMAGE* software (Matsushita, 2023 ▸). The experimental conditions and data-processing parameters are summarized in Table 1 ▸.

## 4. Results

**Figure 4:**
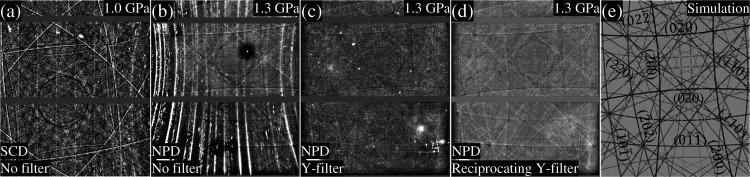
Comparison of hologram images of SrTiO_3_ under various DAC experimental conditions: (*a*) SCD anvils, (*b*) NPD anvils, (*c*) NPD anvils with a Y filter, (*d*) NPD anvils with a reciprocating Y filter, and (*e*) simulated hologram image at 1.3 GPa with a camera length of 63 mm and a lattice constant of *a* = 3.8967 Å (Seto & Ohtsuka, 2022 ▸).

One of the most serious technical problems in high-pressure XFH experiments is that the millimetre-sized single-crystalline diamond (SCD) anvils generate strong pseudo-Kossel lines (Okada & Iwasaki, 1980 ▸; Novelli *et al.*, 2022 ▸). These pseudo-Kossel lines are superimposed on the Kossel lines from the sample, making it difficult to extract the sample’s Kossel lines. Fig. 4 ▸(*a*) shows our experimental observation where the pseudo-Kossel lines completely obscure the Kossel lines of the sample in the DAC.

NPD anvils are composed of randomly oriented crystalline diamonds with a typical grain size of ∼10 nm, providing a background without the pseudo-Kossel lines. Additionally, NPD anvils offer good optical transmission and greater hardness compared with SCD anvils, making them well suited for high-pressure experimental setups (Irifune *et al.*, 2003 ▸; Ishimatsu *et al.*, 2012 ▸). Figs. 4 ▸(*b*)–4(*d*) show hologram images of SrTiO_3_ obtained using NPD anvils. The absence of pseudo-Kossel lines by NPD anvils is recognized in Fig. 4 ▸(*b*), and the sample’s hologram pattern can be observed. However, the polycrystalline structure of NPD alternatively produces Debye–Scherrer rings as background noise. Furthermore, the elimination of pseudo-Kossel lines reveals that the Be gasket generates spotty noise owing to diffraction from its polycrystalline structure. Therefore, further optimization of the experimental setup is necessary to remove these undesired diffraction patterns.

The holograms from the sample are two to three orders of magnitude weaker than the diffraction signals from the NPD anvils and the Be gasket. To suppress the elastic scattering, a foil filter is typically employed in XFH experiments (Busetto *et al.*, 2000 ▸). Y foil is an appropriate choice for measuring Sr *K*α emission (14.165 keV) as the hologram image. As shown in Fig. S1 of the supporting information, a 125 µm-thick Y foil reduces the elastic scattering at 17.391 keV by two orders of magnitude, while the Sr *K*α holograms are only modestly affected. Fig. 4 ▸(*c*) shows a hologram image after being filtered by the Y foil. The filter was positioned behind the DAC window to cover the entire XFH signal. Although the diffraction patterns from the NPD and Be gasket were eliminated, the contrast of the hologram in Fig. 4 ▸(*c*) is lower than that in Fig. 4 ▸(*b*) owing to an inhomogeneous background caused by minor scratches and surface modulations on the filter. To improve the flatness of the filter as much as possible, a crank mechanism was installed [see Fig. 1 ▸ and the inset of Fig. 2 ▸(*c*)]. During the measurement, the Y filter was reciprocated over a range of ±1 mm along both vertical and horizontal directions in the plane perpendicular to the emitted X-rays. The frequency of the reciprocation was 1 min. It was confirmed that the reciprocating filter always covered the entire DAC window. Consequently, a clear hologram pattern with a smooth background was obtained, as shown in Fig. 4 ▸(*d*). This reciprocating motion effectively ensured the flatness of the Y-filter surface and maximized background smoothness. Notably, the high-pressure hologram pattern is in good agreement with the simulation calculated using the software *ReciPro* [Fig. 4 ▸(*e*)] (Seto & Ohtsuka, 2022 ▸).

**Figure 5:**
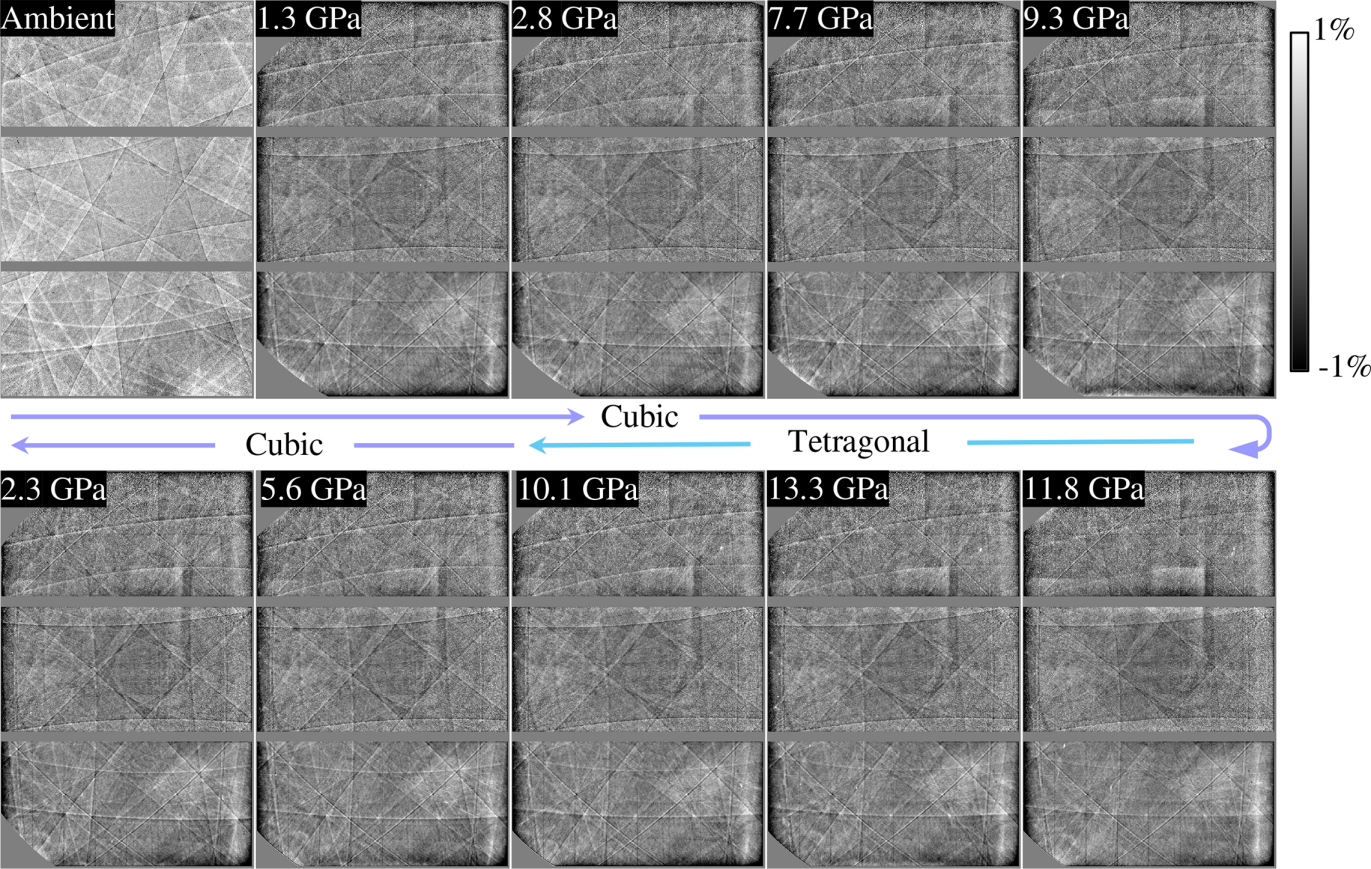
Pressure dependence of the flattened 2D holograms. Holograms, after appropriate background subtraction, are shown. The hologram at ambient pressure was collected without the DAC.

Using the optimized experimental setup, we successfully acquired hologram patterns at various pressures, as shown in Fig. 5 ▸. Data at ambient pressure were collected without the DAC. Under high pressure, the Kossel lines in the hologram images exhibit lower intensity compared with those at ambient pressure, primarily owing to the smaller sample size within the DAC and the ineligible X-ray absorption by the Y filter. Nevertheless, the quality and resolution of the data are sufficient for subsequent processing. Pressures up to 13.3 GPa were achieved, exceeding the phase-transition pressure of SrTiO_3_ (∼10 GPa) (Guennou *et al.*, 2010 ▸). Kossel lines remain visible up to 13.3 GPa owing to the excellent hydrostatic conditions maintained by the helium pressure-transmitting medium. The sample with a thickness of 13 µm was sufficiently thin to avoid direct compression by the anvils.

**Figure 6:**
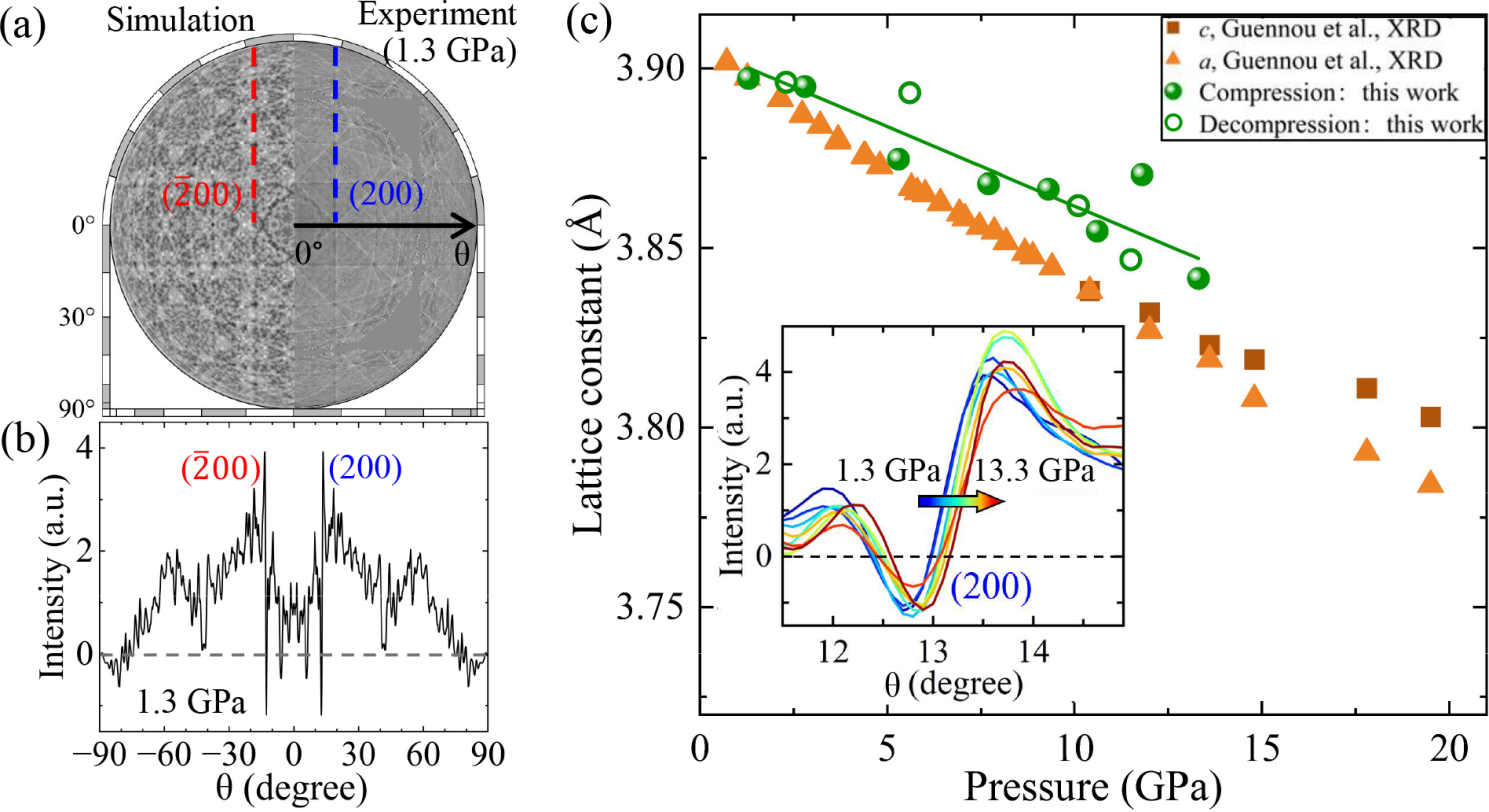
(*a*) Comparison of spherical hologram images between simulation (left) and experiment at 1.3 GPa (right). (*b*) Kossel line profile as a function of scattering angle θ at 1.3 GPa. (*c*) Variation of lattice constants with pressure. The inset shows the shift of the profile around the (200) Kossel line with pressure.

As shown in Fig. 3 ▸, the 2D hologram images were projected onto the Ewald sphere and expanded to cover the entire spherical surface using appropriate symmetry operations. The pixel size was optimized by comparing the quality of the resulting atomic images, with a size of 30 pixels chosen for this experiment. Blank regions in the data, such as gaps between the three CCD devices and the top-left and bottom-left corners of the original 2D images, were assigned a value of zero to avoid artificial noise. Holograms on the entire spherical surface were generated by applying symmetry operations based on the cubic structure: fourfold symmetry about the *k*_*x*_, *k*_*y*_ and *k*_*z*_ axes, and mirror symmetry with respect to the *k*_*x*_*k*_*y*_, *k*_*y*_*k*_*z*_ and *k*_*z*_*k*_*x*_ planes. Fig. 6 ▸(*a*) compares the experimental hologram with a theoretical calculation based on an atomic cluster of cubic SrTiO_3_ with a diameter of 50 Å. The close agreement between the experimental and simulated holograms indicates that the SrTiO_3_ holograms under high pressure were accurately extracted by our data-processing procedures. Using the same methods, we constructed spherical hologram images for all measured pressures, as shown in Fig. S2.

To quantify the evolution of the crystal structure during compression and decompression, shifts of the Kossel lines were measured as a function of the scattering angle, θ. Fig. 6 ▸(*b*) shows a cross-sectional profile along the (*h*00) plane at 1.3 GPa, where the dip and hump at θ = 12.97° and −12.97° correspond to the (200) and 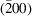 Kossel lines, respectively. The inset in Fig. 6 ▸(*c*) shows enlarged profiles around the (200) Kossel lines at selected pressures, showing a shift towards higher θ angles with increasing pressure, indicative of lattice compression. The *d* spacing of the (*hkl*) planes and the lattice constant, *a*, can be calculated using the criterion 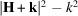 = 0. This corresponds to the singularity in the function 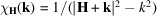, where 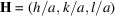 are reciprocal-space vectors, 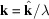 is the radiation wave vector and 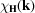 denotes the holograms of the atomic planes associated with 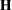 (Hayashi & Korecki, 2018 ▸). The experimental profile of the (200) plane is proportional to the 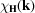 function at 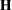 = (2/*a*, 0, 0). Taking into account broadening due to experimental resolution, we define the singularity point as the θ angle at which the profile crosses zero [*i.e.*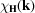 = 0]. The main panel of Fig. 6 ▸(*c*) shows pressure dependence of the determined lattice constant. The lattice constant decreases linearly from 3.897 Å at 1.3 GPa to 3.842 Å at 13.3 GPa, evaluated from θ values of the (200) Kossel line. The measured compressibility gives marginally larger lattice constants and their deviations from the fitted line compared with the reported compressibility (Guennou *et al.*, 2010 ▸), which we attribute to an asymmetric cross-sectional profile relative to the zero line [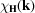 = 0] caused by residual positive background not fully subtracted during processing.

**Figure 7:**
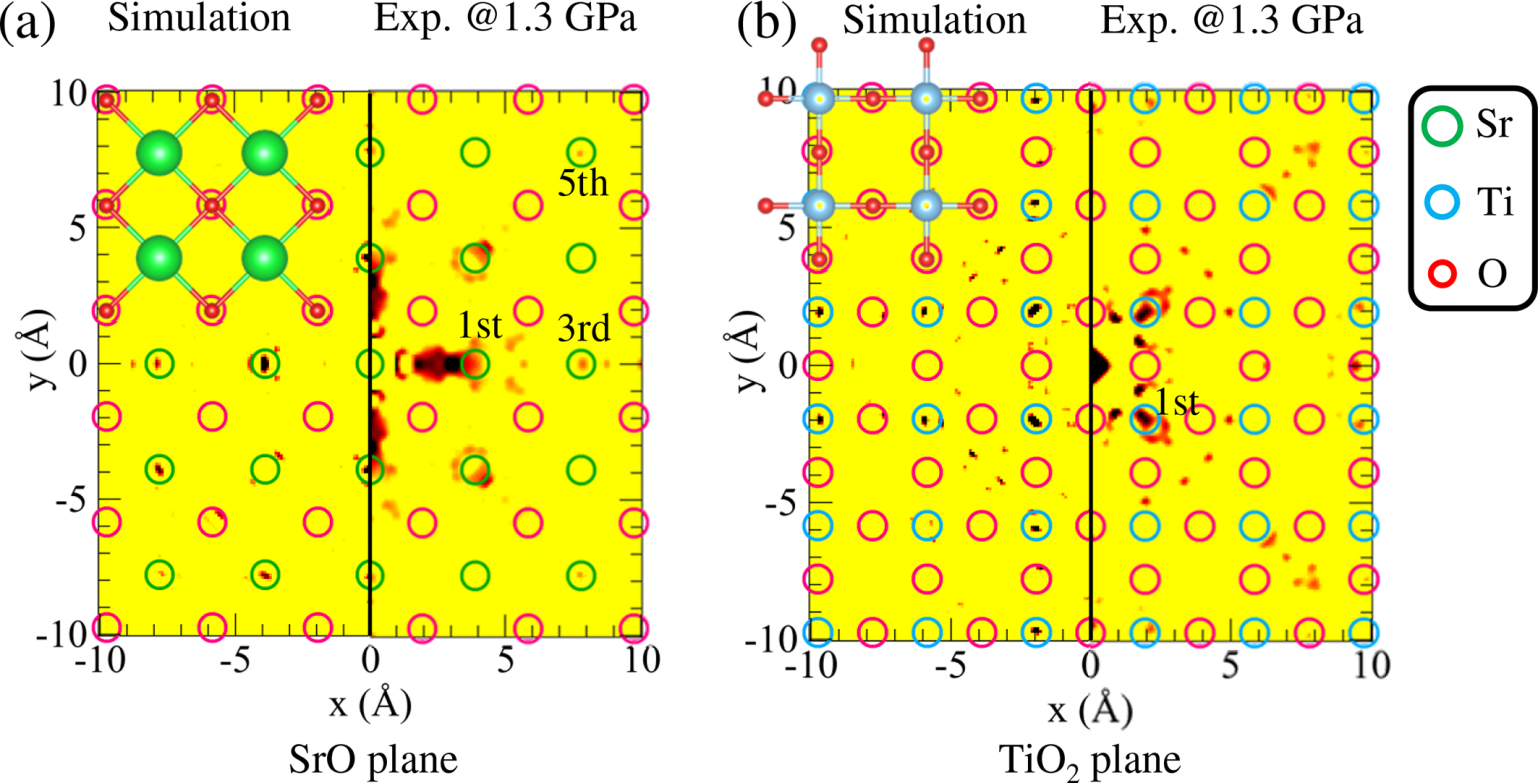
Reconstructed 2D images of SrTiO_3_ at 1.3 GPa: (*a*) SrO plane and (*b*) TiO_2_ plane. Additional atomic images at other pressures are shown in Fig. S4. Circles present the expected atomic positions at 1.3 GPa.

Fig. 7 ▸ presents a comparison of the reconstructed atomic images in the SrO and TiO_2_ planes between theoretical (left) and experimental results at 1.3 GPa (right). Barton’s algorithm was used to reconstruct the atomic images from the spherical holograms (Barton, 1988 ▸). Circles indicate the expected positions of atoms based on a cubic model with a lattice constant of 3.897 Å at 1.3 GPa (Guennou *et al.*, 2010 ▸). In these images, the positions of the first, third and fifth nearest-neighbour Sr atoms are visible [Fig. 7 ▸(*a*)], as well as those of the first nearest-neighbour Ti atoms [Fig. 7 ▸(*b*)]. As the image intensity is proportional to the atomic number, images of the lighter oxygen atoms are absent. Several images observed in the interstitial regions are likely artefacts, which commonly occur in normal-mode holograms using single-energy X-rays (Hayashi *et al.*, 2012 ▸). Reconstructed atomic images for all measured pressures are provided in Figs. S3 and S4. In contrast to the Kossel lines, systematic changes in atomic positions due to lattice compression or structural transitions were hardly observed. SrTiO_3_ undergoes a lattice compression of Δ*a*/*a* ≃ 0.02 at 13.3 GPa (Guennou *et al.*, 2010 ▸; Parisiades *et al.*, 2016 ▸), a change that is considerably small to be resolved within the typical spatial resolution (∼0.5 Å) of the reconstructed atomic images (Hayashi *et al.*, 2012 ▸).

## 5. Discussion

Clear holograms and atomic images of SrTiO_3_ under high pressures have been successfully obtained; therefore, it is worth simulating the modifications in hologram patterns induced by the structural transition to the tetragonal phase. As reported by the X-ray diffraction measurement (Guennou *et al.*, 2010 ▸), the *c*/*a* ratio is ∼1.001 at 13.3 GPa, corresponding to a scattering-angle split of Δθ ≃ 0.014°. This split is considerably small to be resolved by the sharpness of the experimental Kossel lines; however, it is anticipated that at pressures around 30 GPa, where the *c*/*a* ratio and Δθ increase by at least an order of magnitude, the split will become discernible. To support our speculation, simulated Kossel lines of the tetragonal phase are shown in Fig. S5. We constructed a tetragonal phase structure according to the report by Guennou *et al.* (2010 ▸). At a pressure near 13.3 GPa, splits of Kossel lines are difficult to see in the simulated results, which is consistent with our experimental results. However, as the pressure increases, the *c*/*a* ratio increases, and splits of Kossel lines with different lattice orientations can be clearly seen at a pressure near 30 GPa. Therefore, it is feasible to quantify the anisotropic lattice contraction of SrTiO_3_ by the shift and split of the Kossel line under higher-pressure conditions.

In the reconstructed images at high pressures, atomic images at the distant sites are absent or displaced from their expected positions, and pronounced artefacts appear in the interstitial regions. These issues are primarily attributed to the use of a single-energy hologram for reconstruction and the limited statistical precision of the fluorescence signal. Additional artefacts may also arise from non-uniform background noise in the holograms, a consequence of imperfect background removal during both the measurements and data processing. Addressing these challenges will require further advancements in elastic scattering cut-off filters, 2D detectors, background-correction methods and data-processing methods (Matsushita *et al.*, 2018 ▸).

Finally, we emphasize that the distribution of Kossel lines provides valuable insights into pressure-induced structural changes around the X-ray absorbing element. In this study, we have presented that lattice compression can be quantified by tracking shifts in the Kossel lines. If splits or the appearance/disappearance of Kossel lines occur, these would provide direct evidence of changes in crystallographic symmetry around the X-ray absorbing atom (Nakashima *et al.*, 2024 ▸). Compared with the difficulties in reconstructing atomic images, the analysis of Kossel lines is less affected by the low signal-to-noise ratio under high pressure. As shown here, the effective removal of background noise now permits quantitative analysis of Kossel lines under high pressure. Therefore, we propose that the method developed in this study presents a significant advantage for using XFH to investigate pressure-induced changes in the local structure around doped atoms and other specific elements, with applications in analysing the crystallographic symmetry around a doped element such as thermoelectric and dielectric materials.

## 6. Conclusions

We successfully combined XFH with a DAC to perform high-pressure measurements. Using a normal-mode geometry and focused X-rays, Sr *K*α holograms of a SrTiO_3_ single crystal were recorded, yielding uniform XFH images at pressures up to 13.3 GPa. The key technical advancements in this study include: (1) replacing SCD anvils with NPD anvils to eliminate pseudo-Kossel lines arising from the diamond crystal structure, (2) compressing a thin SrTiO_3_ crystal in a helium pressure medium to maintain high lattice quality under pressure, and (3) employing a Y foil as a high-energy cut-off filter to remove elastic scattering noise from both the NPD anvils and the gasket. Shifts in the Kossel lines were used to determine the variation of lattice constants with pressure. Moreover, real-space images of the atomic structures, reconstructed from the Sr *K*α holograms, were acquired at various pressures. This work demonstrates the applicability of XFH under high-pressure conditions as a novel method for visualizing pressure-induced changes in 3D atomic structures. In particular, the analysis of Kossel lines provides a useful method for investigating symmetry breaking and lattice compression in the local structure surrounding specific elements under high pressure.

## Supplementary Material

Experimental data supplement. DOI: 10.1107/S1600577525005284/ok5136sup1.pdf

## Figures and Tables

**Table 1:** Experimental conditions and data-processing parameters

Parameter	Value	Parameter	Value
Incident X-rays	17.391 keV	DAC culet	Ø450 µm
Size of X-ray beam	1.13 µm × 0.68 µm	Size of chamber	Ø220 µm × 47 µm
Incident angle	5°	Size of sample	70 µm × 70 µm × 13 µm
Camera length	63 mm	Size of Y filter	50 mm × 50 mm × 0.125 mm
Pixel size of detector	100 µm × 100 µm	Area of detector	78 mm × 122 mm
Polar angle resolution	0.5 pixels	Calculation resolution	0.5 pixels

## Data Availability

The data that support the findings are available from the corresponding author on reasonable request.
