# Supplementary material for: X-ray fluorescence holography under high-pressure conditions
Source: J Synchrotron Radiat. 2025 Jul 18;32(Pt 5):1302–9. doi: 10.1107/S1600577525005284 (PMC12416426; doi:10.1107/S1600577525005284)
Supplement: Supplementary file 1 [file s-32-01302-sup1.pdf]

JOURNAL OF SYNCHROTRON RADIATION

Volume 32 (2025)

Supporting information for article:

X-ray fluorescence holography under high-pressure conditions

Xinhui Zhan, Naoki Ishimatsu, Koji Kimura, Naohisa Happo, Halubai Sekhar, Tomoko Sato, Nobuo Nakajima, Naomi Kawamura, Kotaro Higashi, Oki Sekizawa, Hirokazu Kadobayashi, Ritsuko Eguchi, Yoshihiro Kubozono, Hiroo Tajiri, Shinya Hosokawa, Tomohiro Matsushita, Toru Shinmei, Tetsuo Irifune and Koichi Hayashi

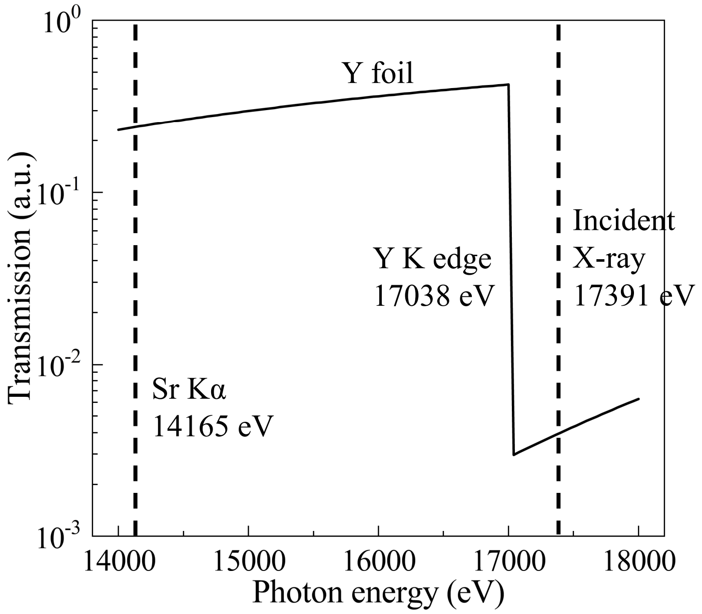

Figure S1: X-ray transmission of a 125 μm thick Y foil at energies around the Y K edge.

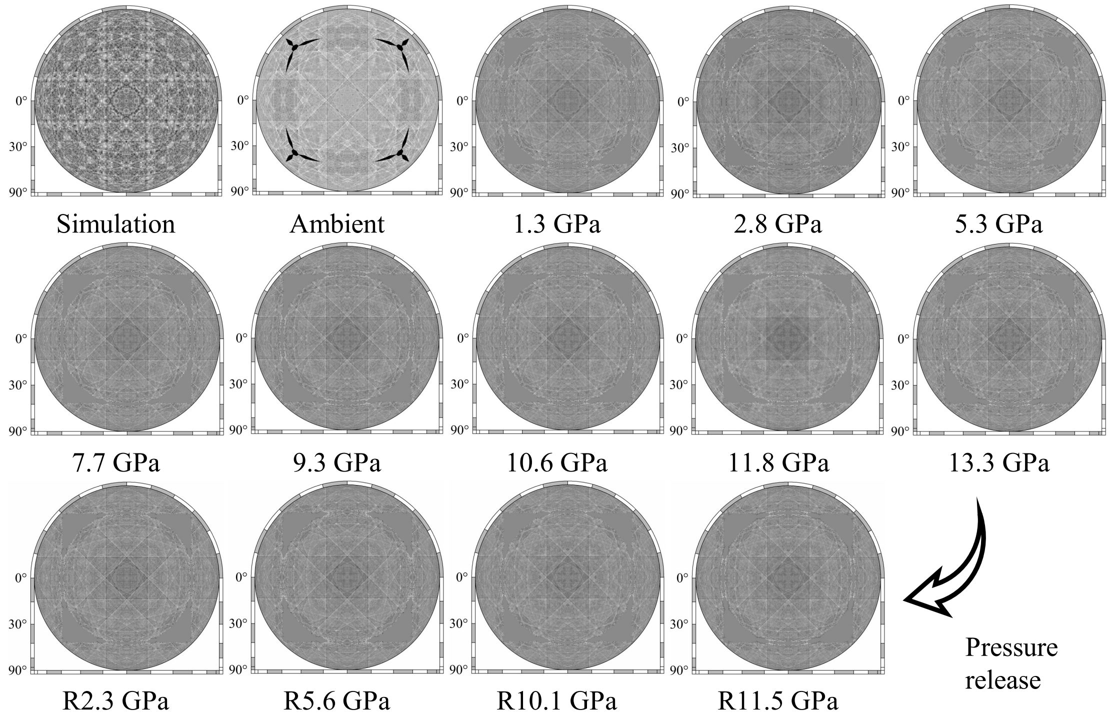

Figure S2: Orthographic projection of Sr Kα hologram patterns, projected onto the Ewald sphere in k-space, under various pressures.

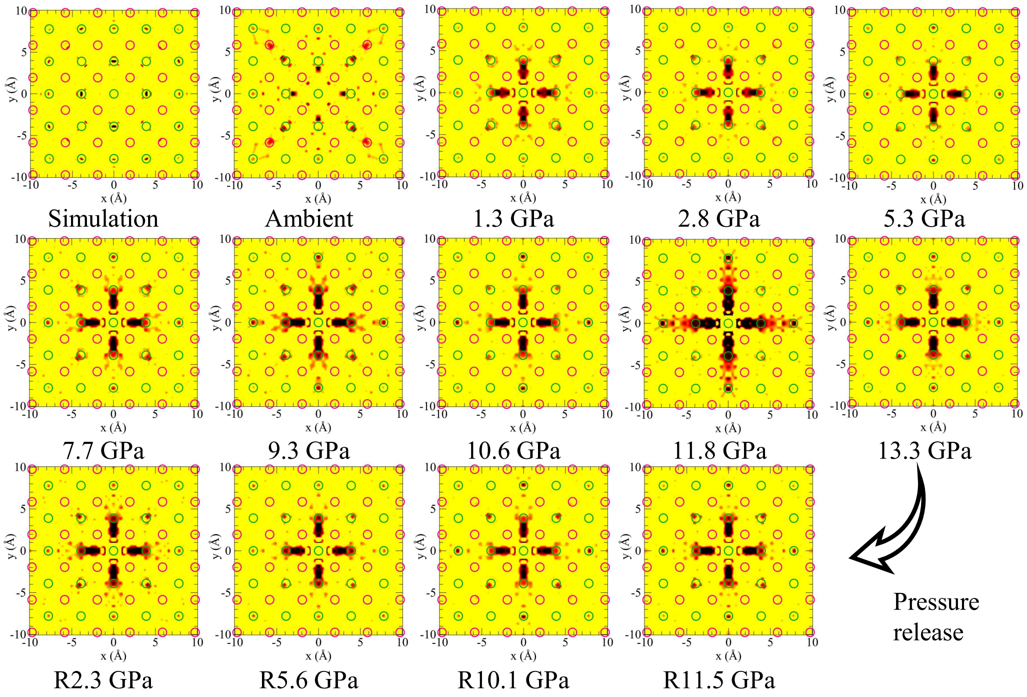

Figure S3: Reconstructed 2D atomic arrangement in the SrO plane of $SrTiO_3$. All circles present the expected atomic positions at 1.3 GPa. The fixed position of the circles enables us to find relative shifts of the atomic images with pressure.

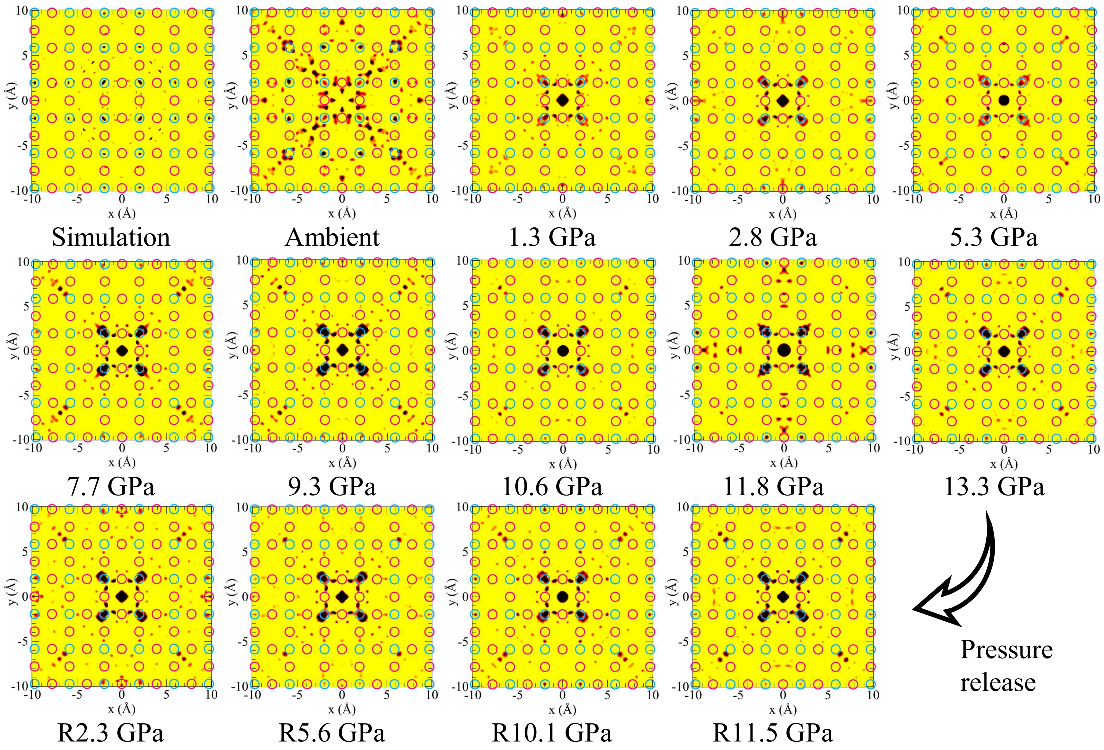

Figure S4: Reconstructed 2D atomic arrangement in the TiO_2 plane of $SrTiO_3$. All circles present the expected atomic positions at 1.3 GPa. The fixed position of the circles enables us to find relative shifts of the atomic images with pressure.

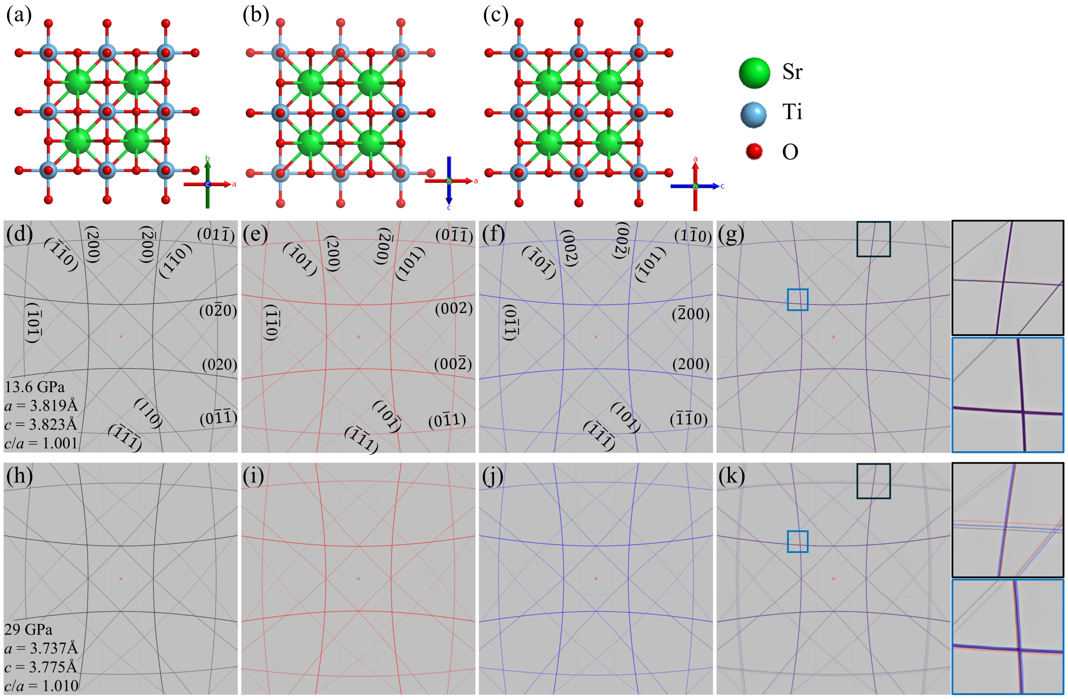

Figure S5: (a)-(c): Atomic structure of the tetragonal $SrTiO_3$ with three crystal orientations used for the simulation of Kossel lines. (d)-(k): Simulated Kossel lines for the tetragonal phase at (d)-(f) 13.6 GPa and (h)-(j) 29 GPa corresponding to the crystal orientations of (a)-(c). Panels (g) and (k) show experimentally expected images of the hologram at (g) 13.6 GPa and (k) 29 GPa. These images are obtained by superimposing the Kossel lines of panels (d)-(f) and (h)-(j), respectively. To clarify the split of Kossel lines, enlarged images of the selected areas are shown aside.

Software ReciPro was utilized to examine the split of Kossel lines of tetragonal $SrTiO_3$. As shown in Figs. S5(a)-S5(c), to simplify our model, only lattice contraction was assumed under high pressure. Lattice constants of the tetragonal phase were taken from a paper (Guennou *et al.*, 2010). This simulation assumed an equal probability for the formation of tetragonal phases with three different crystal orientations, where the tetragonal c-axis aligns parallel to one of the three cubic a-axes. Experimentally expected hologram images of the tetragonal phase can be obtained as Figs. S5(g) and S5(k) by superimposing the Kossel lines of the three crystal orientations. Figure S5(g) demonstrates that splits of Kossel lines are small and difficult to observe for $c/a \sim 1.001$ at 13.6 GPa. However, at higher pressure, e.g. Fig. S5(k) at 29 GPa, splits of Kossel lines with different crystal orientations can be seen due to lager c/a ratio ($c/a \sim 1.010$).

Reference

Guennou, M., Bouvier, P., Kreisel, J. & Machon, D. (2010). Phys. Rev. B, 81, 054115. https://link.aps.org/doi/10.1103/PhysRevB.81.054115
